# Exploring the potential of phyllosilicate minerals as potassium fertilizers using sodium tetraphenylboron and intensive cropping with perennial ryegrass

**DOI:** 10.1038/srep09249

**Published:** 2015-03-18

**Authors:** Ting Li, Huoyan Wang, Jing Wang, Zijun Zhou, Jianmin Zhou

**Affiliations:** 1 State Key Laboratory of Soil and Sustainable Agriculture, Institute of Soil Science, Chinese Academy of Sciences, Nanjing 21008, China; 2 College of Resources and Environment, Sichuan Agricultural University, Wenjiang 611130, China; 3 University of Chinese Academy of Sciences, Beijing 100049, China

## Abstract

In response to addressing potassium (K) deficiency in soil and decreasing agricultural production costs, the potential of K-bearing phyllosilicate minerals that can be directly used as an alternative K source has been investigated using sodium tetraphenylboron (NaTPB) extraction and an intensive cropping experiment. The results showed that the critical value of K-release rate and leaf K concentration was 3.30 g kg^−1^ h^−1^ and 30.64 g (kg dry matter)^−1^, respectively under the experimental conditions. According to this critical value, the maximum amount of released K that could be utilized by a plant with no K deficiency symptoms was from biotite (27.80 g kg^−1^) and vermiculite (5.58 g kg^−1^), followed by illite, smectite and muscovite with 2.76, 0.88 and 0.49 g kg^−1^, respectively. Ryegrass grown on phlogopite showed K deficiency symptoms during the overall growth period. It is concluded that biotite and vermiculite can be directly applied as a promising and sustainable alternative to the use of classical K fertilizers, illite can be utilized in combination with soluble K fertilizers, whereas muscovite, phlogopite and smectite may not be suitable for plant growth. Further field experiments are needed to assess the use of these phyllosilicate minerals as sources of K fertilizer.

Potassium (K) is considered a major nutrient element together with nitrogen (N) and phosphorus (P) for plant growth. However, in contrast to N and P, K fertilizers are applied at a much lower rate, and less than 50% of the K removed by crops is replenished^1^. Therefore, large agricultural areas of the world are reported to be deficient in available K^2^,^3^,^4^. Potassium deficiency in China is partly because the majority of K fertilizer in China is imported and therefore cost prohibitive and partly because farmers are not aware of the economic benefit of applying K fertilizer^5^. Thus, investigation of K-bearing minerals and rocks that can be directly used in soil as an alternative potassium source is an important step toward addressing K deficiency in soil and decreasing agricultural production costs.

The most abundant sources of K-bearing minerals and rocks that can be used as alternative K fertilizers include K-feldspars and phyllosilicate minerals. K-feldspars are commonly found in the silt and sand fractions of young to moderately developed soils, representing various soil-forming conditions^6^,^7^. However, it is normally assumed that the weathering of K-feldspars is very slow, and that their addition to soil will not be beneficial for crop growth^8^,^9^,^10^. The primary K-bearing phyllosilicate minerals include micas and their weathering products. Micas are most common in young, less weathered soils^11^. Weathering of micas may produce secondary minerals that also represent potential sources of K in the soil. These include a range of micaceous clay minerals such as illite, vermiculite and smectite^12^. Selecting the best phyllosilicate minerals to be used as fertilizers does not depend on the absolute K content, but depends primarily on the rate at which K can be released into more labile forms that can be utilized by plants^13^,^14^,^15^,^16^. For the mineral to be a useful source of K on a short-term basis, its K should be at least as plant available as the native K in the soil.

To estimate the amount of K in soils that is available to plants, the K-release capacity is often determined using sodium tetraphenylboron (NaTPB)^17^,^18^,^19^,^20^,^21^,^22^. Extracting K from soil using NaTPB can mimic the action of plant roots by depleting soil-solution K^+^ as KTPB and cause further release of interlayer K^+19^, thus, the easily extractable K is released first, then the recalcitrant interlayer K is released gradually^23^. High correlations have been obtained between the K extracted from soil using NaTPB solutions and the amount of K taken from the soils by cropping^18^,^19^,^20^,^21^. However, little attention has been given to the K-bearing phyllosilicate minerals, the relationship between the K-release capacities of K-bearing phyllosilicate minerals under cropping, and whether their K release is sufficient for the plant K requirements.

Intensive cropping with ryegrass in pots is a frequently used method to assess K bioavailability in soil or minerals^13^,^24^. The aims of our research were: (1) to investigate the release capacity of K from different K-bearing phyllosilicate minerals using NaTPB solutions, (2) to study the effects of K release from K-bearing phyllosilicate minerals on perennial ryegrass (*Lolium perenne* L.) grown in a quartz sand and (3) to assess the K-supply ability of phyllosilicate minerals.

## Results

### Properties of sample phyllosilicate minerals

Table 1 shows the complete chemical analysis and selected properties for phyllosilicate minerals. The sample minerals were 2:1 phyllosilicates with a charge imbalance that is satisfied by a tightly held, nonhydrated interlayer cation. The interlayer cation was mainly the K^+^ ion in the K-bearing minerals. The 2:1 layer was composed of an octahedral sheet between two tetrahedral sheets. Thus, the main chemical composition of the tested phyllosilicate minerals were silicon dioxide (SiO_2_) and aluminium oxide (Al_2_O_3_) as shown in Table 1. Muscovite, phlogopite and illite contained greater K_2_O (9.18%, 10.02% and 9.68%, respectively) than biotite, vermiculite and smectite (6.73%, 2.26% and 3.92%, respectively). Biotite contained more slow available K (10,399 mg kg^−1^) than the other sample minerals. In general, biotite and illite had similar cation exchange capacities (CEC) (7.25 and 6.00 cmol kg^−1^, respectively), while muscovite, phlogopite, vermiculite and smectite exhibited different CEC (2.30, 1.75, 47.05 and 14.25 cmol kg^−1^, respectively). The sample minerals contained 0.12–20.38% MgO (an additional important plant nutrient) and 0.04–1.62% CaO.

### Cumulative K release and the release pattern

Cox and Joern^23^ found that K release from the interlayer of phyllosilicate clay minerals in all soils was almost complete after 96 h of incubation with NaTPB. Therefore, K release was evaluated for the period ranging from 5 min to 96 h in our study. The results showed that cumulative K release increased with time for all of the phyllosilicate minerals. The cumulative release of K ranged from 0.20–5.73 g kg^−1^ at 5 min to 1.31–48.30 g kg^−1^ at 96 h. The maximum amount of K was released by biotite (48.30 g kg^−1^), whereas the minimum K was released by muscovite and smectite (1.69 and 1.31 g kg^−1^) at 96 h. The cumulative release of K from illite and vermiculite was an average of four times greater than the cumulative release of K from phlogopite at 96 h. The amount of K released by NaTPB from the sample minerals ranged from 1.8–71.8% of their total K.

**Figure 1:**
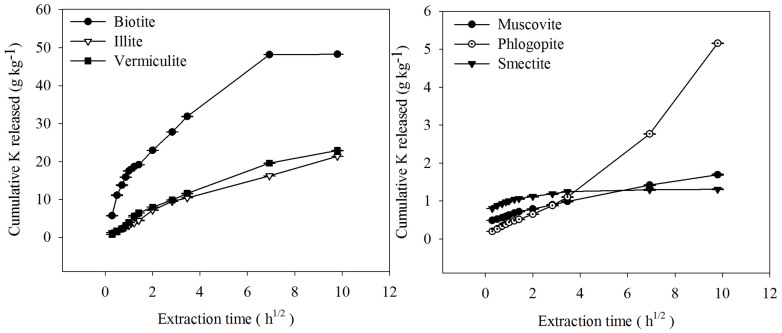
Potassium release in 0.2 M NaTPB over time for different K-bearing minerals.

The K release values were plotted against time to observe the K release pattern (Figure 1). On average, there was a faster K release during the first 2 h and thereafter K release slowed down considerably except for phlogopite, which showed a continuous trend of fast release during the extraction time. The percent of K released from the sample minerals in 2 h ranged from 20–82% of the total K release, although phlogopite released only 10% of the total K in 2 h. Therefore, the curve plotted in Figure 1 has one part representing an initial rapid K release and a second part representing a slower K release.

### Dry matter yield of ryegrass with K supply from K-bearing minerals

**Figure 2:**
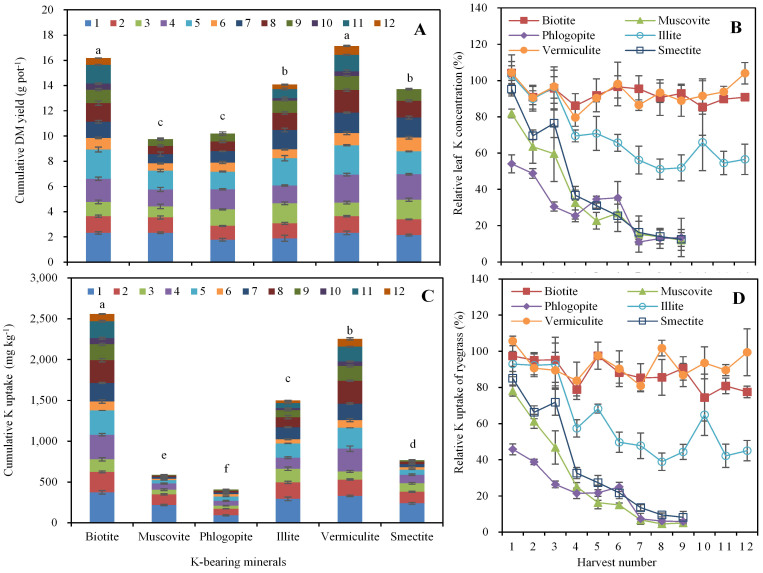
Growth of ryegrass with K supply from K-bearing minerals. (A) depict the cumulative dry matter (DM) yield of ryegrass, (B) show the relative leaf K concentration of ryegrass, (C) and (D) describe the cumulative and relative K uptake of ryegrass. Bars indicate standard error. Values presented are means ± standard error (n = 4). Different small letters denote significant differences (*P* < 0.05) among the K-bearing minerals.

The different phyllosilicate minerals supported large differences in dry matter (DM) yield and K concentration in ryegrass (Figure 2A). As indicated by the cumulative DM yield from all 12 or all 9 crops, the ryegrass only grew well in the vermiculite treatment, which was the most K-sufficient mineral and it provided a significantly higher DM yield of 17.14 g pot^−1^, followed by biotite with 16.19 g pot^−1^. For the treatment with K supplied by illite, the growth of ryegrass was seriously stunted and provided a DM yield of 1.89 g pot^−1^ for the first crop but declined to 0.34 g pot^−1^ for the last crop. Ryegrass could not survive for nine crops without external K when it grew on smectite, muscovite and phlogopite.

### Leaf K concentration in ryegrass

The trends for leaf K concentrations in ryegrass with different treatments was similar to the trends for the cumulative DM yields (Figure 2B). The greatest leaf K concentrations occurred from biotite and vermiculite for the first to the final crop with only a slight decline, followed by illite. Muscovite, phlogopite, illite and smectite showed a significant decline in leaf K concentrations from the first to the final crop.

### Cumulative and relative K uptake by ryegrass

Cumulative and relative K uptake by ryegrass during cropping varied widely between the phyllosilicate minerals (Figure 2C and 2D). The quantity of K uptake in the first ryegrass crop varied from 92.44 to 373.73 mg kg^−1^ between the sample minerals. Greater K uptake was observed in the first three crop harvests, with considerably less uptake in subsequent crops. The cumulative K uptake during the overall period of 12 or 9 crops varied from 408.21 mg kg^−1^ to 2,559.29 mg kg^−1^. In contrast, the cumulative K uptake by ryegrass was significantly higher in biotite, less in vermiculite and illite, and the lowest in smectite, muscovite and phlogopite (Figure 2C). A comparison of the relative uptake of K by ryegrass between phyllosilicate minerals (Figure 2D), showed that biotite and vermiculite were the highest with only a slight decrease of 19.80% and 6.25% from the first to twelfth harvest; illite was intermediate with a high value of 92.94% initially but only 45.07% for the last harvest; whereas muscovite, phlogopite and smectite were the lowest initially and decreased significantly of 73.00%, 39.97% and 76.84%, respectively.

## Discussion

Plots of cumulative amounts of K release showed a discontinuity in slope at 2 h. Therefore, two equations were applied to the segments of the plot for cumulative K release in the reaction time of 0–2 h and 2–96 h. This discontinuity represents different mechanisms controlling the release process as mentioned in the previous section^25^,^26^,^27^,^28^,^29^,^30^. Ghosh and Mukhopadhyay^31^ explained that rapid and slower K release corresponds to the release of external and lattice K, respectively. The release of K from mineral surfaces (exchangeable sites) was easier as Vander Walls (physical absorption) and Lewis forces (chemisorption) were responsible for binding the K to the mineral surface, whereas desorption from the lattice portion of the mineral required more energy, for example lattice energy, to release K^27^.

Different kinetic models were used to describe K release from the minerals for the two segments of the plot (Table 2). For the two segments (0–2 h and 2–96 h), comparisons of values for the coefficient of determination (R^2^) and the standard error of estimate (SE) showed that the parabolic diffusion and power function equations had the greatest determination coefficients (R^2^ = 0.767–0.997 and 0.886–0.998, respectively), the lowest standard error of estimates (SE = 0.01–4.39 and 0.01–3.31 g kg^−1^) and they could describe K release. In addition, the Elovich equation (R^2^ = 0.875–0.991, SE = 0.01–2.59 g kg^−1^) described K release kinetics well, whereas first-order and zero-order equations did not. Other workers have reported similar findings in different types of soils using various methods^32^,^33^,^34^,^35^. However, some researchers found that first-order or zero-order equations might sometimes describe K release^27^,^30^,^36^. Results from different studies in the literature indicate that several equations often describe one reaction satisfactorily based on linear regression analysis, but no single equation best describes every case of K release from soils or minerals. This may be attributed to the nature and properties of soils or minerals and experimental conditions.

The merit of the kinetic equations was established not only by their goodness of fit, but also by the utility and physical significance of the information they provided^23^. The fit of the data to the parabolic diffusion and Elovich equations indicate that the K release was a diffusion process^32^. However, the parabolic diffusion and Elovich equations might be difficult for making well-defined comparisons of the K-supplying power of these minerals according to Cox and Joern^23^. The fit of kinetics data to the power function equation did not suggest any particular release mechanism. However, the parameter (*a*) of the equation was an initial release-rate index that could be a good indicator of the K-supplying power in phyllosilicate minerals^37^.

Table 2 indicates that considerable differences in K-release capacity occurred between the phyllosilicate minerals. Rate constants of the two segments (0–2 h and 2–96 h) derived from the power function equations showed that for these minerals the potential to supply plant available K was probably highest for biotite, followed by vermiculite and illite, and lowest by muscovite and smectite. Information from plant growth index studies using these minerals were required to verify the relationship between the K-supplying power and the rate constants estimated for K release by NaTPB.

**Figure 3:**
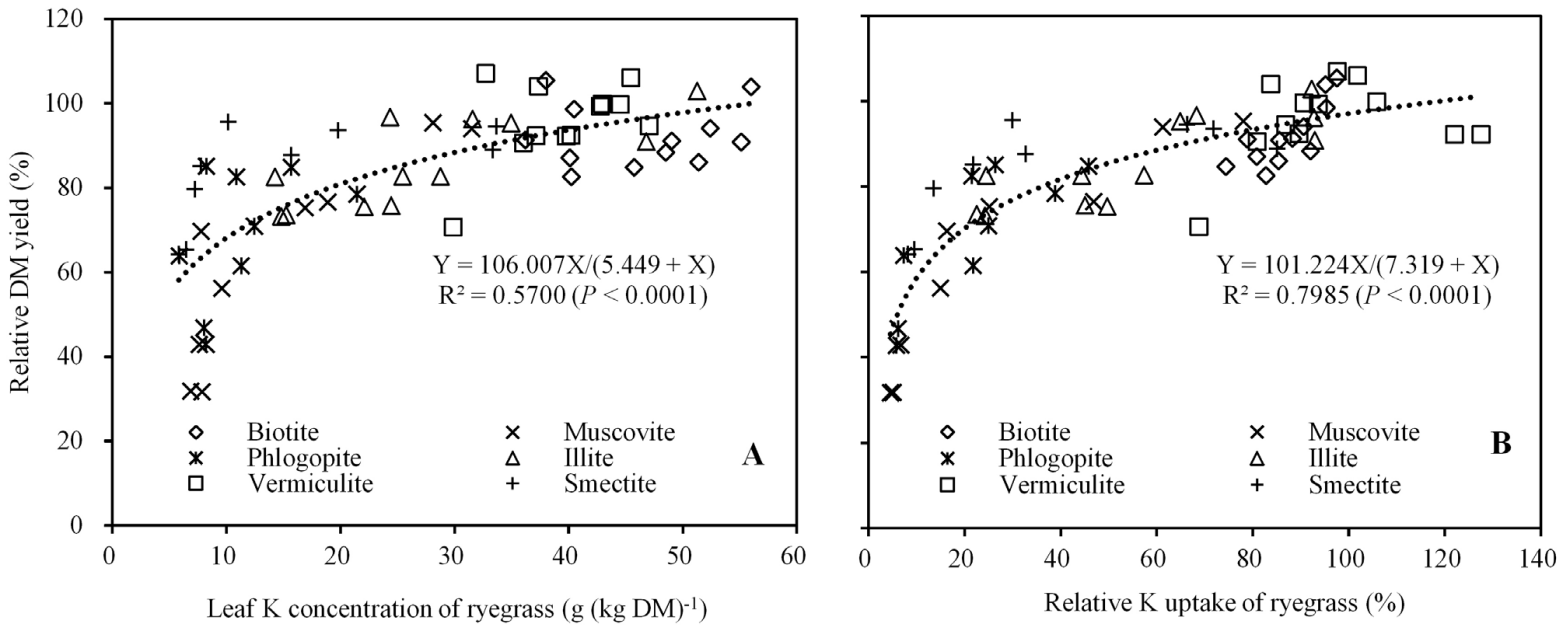
The correlation between relative DM yield and relative leaf K concentration of ryegrass (A) and the correlation between relative DM yield and relative K uptake of ryegrass (B).

The correlation between plant relative DM yield and both leaf K concentration and K uptake by plants are presented in Figure 3A and 3B, respectively. A high and statistically significant correlation (*P* < 0.001) showed that DM yield increased with increasing leaf K concentration and K uptake by plants, indicating K deficiency was the most important growth limiting factor.

The leaf K concentration in ryegrass was a direct index of K-supplying power in phyllosilicate minerals to plants^21^, whereby a lower K concentration indicated K deficiency in ryegrass and significantly reduced the DM yield. A Langmuir equation described the relationship between leaf K concentration and relative DM yield of ryegrass better than the other common equations, evident by its highest determination coefficient (R^2^ = 0.570) (Figure 3A). To judge whether the mineral K supply was deficient or sufficient for plant K uptake under the experimental conditions, the critical K concentration was estimated according to a Langmuir equation. The K concentration of 30.64 g (kg DM)^−1^ was obtained as a critical level to obtain 90% relative DM yield of ryegrass in the current study. The critical level for ryegrass 30.64 g (kg DM)^−1^ approached the range of sufficiency 32–40 g (kg DM)^−1^ determined for field grown wheat^38^, but was much higher than that for ryegrass grown on four soils in East China and on 11 soils in the Midwestern United States which was 18.88 and 19.00 g (kg DM)^−1^, respectively^19^,^21^. The probable cause for different critical leaf K concentrations is related to the growth media. Using the leaf K concentration critical value of 30.64 g (kg DM)^−1^ as a criteria, only biotite and vermiculite could supply sufficient K to satisfy plant requirements from the first to the twelfth crops when K-free nutrient solution was provided. Illite could supply sufficient K for short term plant growth, whereas muscovite, phlogopite and smectite showed K deficiency symptoms from the beginning of plant growth (Figure 3A).

Using the relative DM yields and K uptake as indices for the ease of K release from the minerals (Figure 3B), the following sequence was obtained for the ability of the six phyllosilicate minerals studied to supply K: biotite > vermiculite > illite > smectite > muscovite> phlogopite. However, Norouzi and Khademi^39^ found the sequence for the ability of alfalfa to take up K from different micaceous minerals to be phlogopite > biotite > muscovite. Wentworth and Rossi^18^ showed the sequence for the ability of barley to take up K from different layer silicates were: vermiculite > illite > biotite > phlogopite > muscovite. Differences in sequences obtained for the K uptake by plants from minerals could be caused not only by differing experimental conditions and methods of expressing results, but also by variations in the characteristics of the specific minerals used.

**Figure 4:**
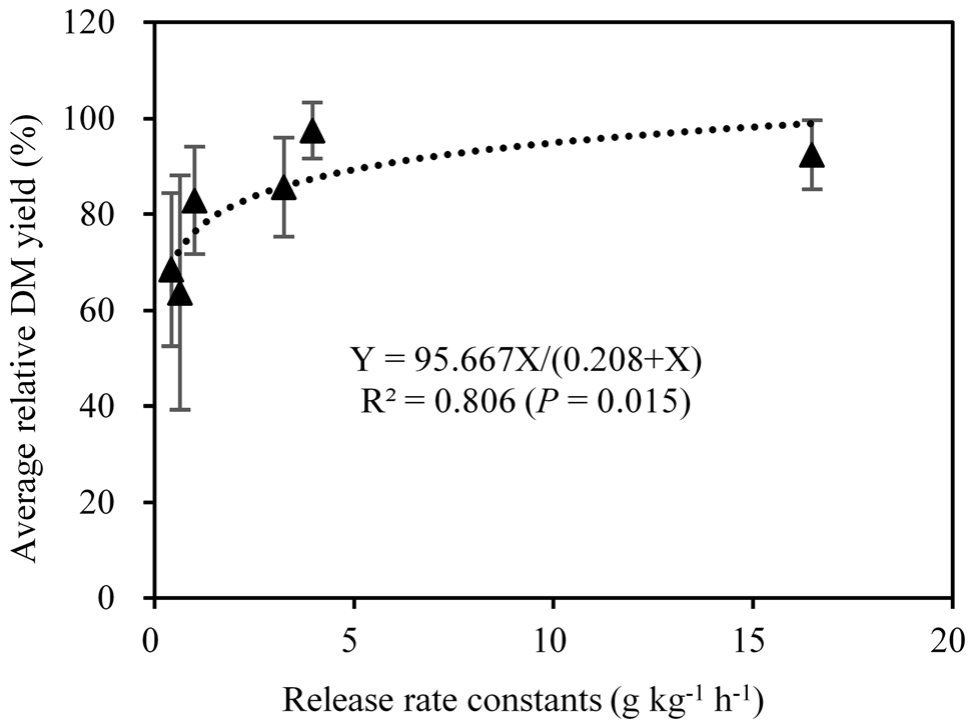
The correlation between rate constants in segment 1 (0–2 h) of the K-release kinetics described by the power function equation and the average relative DM yield for all crop harvests. Bars indicate standard error.

The bioavailability of K in phyllosilicate minerals depends primarily on the rate at which it can be released into more labile forms that can be utilized by plants^13^,^14^,^15^,^16^. Probably the best method to estimate the K-supplying ability of phyllosilicate minerals was the combination of the rate constants of the power equation, which was used to describe the kinetics of K release and the plant growth response on phyllosilicate minerals. Table 3 depicts the correlation coefficients between K-release characteristics and plant indices. The results showed that slow available K and total K of phyllosilicate minerals did not correlate with plant growth indices, similar to the results of Hosseinpur et al.^30^. The amount of K release from phyllosilicate minerals by NaTPB and the release-rate constants of the power function equation in the two segments (0–2 h and 2–96 h) were both significantly correlated with plant indices, and the correlation coefficients of segment 1 (0–2 h) was higher than segment 2 (2–96 h). Thus, the average values of relative DM yield for all crop harvests versus the rate constants of the K-release kinetic of the power function equation in segment 1 (0–2 h) were plotted in Figure 4. The K release rate of 3.30 g kg^−1^ h^−1^ was obtained as the critical level required to obtain 90% average relative DM yield of ryegrass in this study. Using this value as a criterion, the maximum amount of released K that could be utilized by a plant and for the plant to show no K deficiency symptoms was from biotite (27.80 g kg^−1^) and vermiculite (5.58 g kg^−1^), which accounted for 41.3% and 14.2% of the total K in these two minerals, respectively; followed by illite (2.76 g kg^−1^), smectite (0.88 g kg^−1^) and muscovite (0.49 g kg^−1^), which occupied 2.9%, 3.9% and 0.5% of the total K in these three minerals, respectively. Whereas, ryegrass grown on phlogopite showed K deficiency symptoms during the overall period of growth. It is concluded that biotite and vermiculite can be directly applied as a promising and sustainable alternative to the use of classical K fertilizers; illite can be utilized in combination with soluble K fertilizers, whereas muscovite, phlogopite and smectite may not be suitable for plant growth. Further field experiments are needed to assess the applicability of these phyllosilicate minerals as sources of K fertilizer.

## Methods

### Phyllosilicate minerals

The phyllosilicate minerals tested in the series of trials are listed in Table 1. Mica samples including biotite, muscovite and phlogopite, and samples of vermiculite and smectite were obtained from Hebei Province in China. The illite was from Shanxi Province in China. The chemical composition of each mineral sample was analysed using X-ray fluorescence (XRF), the slow available K was determined by boiling with 1 M nitric acid (HNO_3_), and the CEC was determined using 1 M sodium acetate (NaOAc) at pH 7^40^. The samples were ground and sieved to obtain a particle size of <60 μm. To exclude the soluble and exchangeable K contribution to the plants, the exchangeable surfaces of the minerals were saturated with Ca using 0.5 M CaCl_2_ solution. Saturated samples were washed with distilled water several times to remove the excess CaCl_2_ and then dried and used in the pot experiment.

Quartz sand supplied from Anhui Province with a particle size of >60 μm was used as the major plant growth medium. It consisted of almost pure SiO_2_ and was free of clay minerals or other substances. To prepare the quartz sand for the pot experiment, it was washed with 0.5 M HCl, rinsed with deionized water and oven dried.

### K-release capacity

The procedure for using NaTPB to extract K from phyllosilicate minerals was similar to that described by Cox et al.^19^ and Wang et al.^21^. Samples of 0.5 g of minerals, in triplicate, were weighed into 50 mL centrifuge tubes, and 3 mL of extractant (0.2 M NaTPB and 0.01 M EDTA) was added. After shaking at 200 rpm for each incubation period (0.08, 0.25, 0.5, 0.75, 1, 1.5, 2, 4, 8, 12, 48 and 96 h), 25 mL of quenching solution (0.5 M NH_4_Cl and 0.14 M CuCl_2_) was added to each tube to stop the extraction of soil K. The tubes were then heated in boiling water for 1 h to dissolve the KBPh_4_ precipitate. The supernatant was separated by centrifugation at 5000 rpm for 10 min, then diluted with deionized water and K was measured with a flame photometer (Model HG-5, Beijing detection instrument Ltd.) using an internal standard procedure employing 0.003 M LiCl.

The extracted K was plotted against time and the result was fitted to various mathematical models including the first-order equation, zero-order equation, Elovich equation, parabolic diffusion equation and the power function equation^32^,^41^. They were tested using least square regression analysis for their applicability to describe K-release studies. The coefficient of determination (R^2^) and the SE for each of the equations were calculated. These equations were expected to be of help in describing the possible mechanism of K release from minerals and to calculate the distinctive release-rate constants. The forms of each equation used in this study are described below (Table 4).

### Pot experiment

The pot experiment was carried out under greenhouse conditions. Pots were filled with 600 g of a 1:1 mixture by weight of mineral sample to quartz sand. There were two K treatments with four replicates, one in which no K fertilizer was applied throughout the experimental period and one in which K fertilizer was applied. In the K application treatment, K was applied after each plant harvest in a liquid form in quantities equivalent to the K that was removed by the plants in the preceding cutting. Fifty seeds of ryegrass were cropped in each pot. All plants were supplied with N, P, Ca, Mg and micronutrients at sufficient rates and watered with deionized water every 1–2 days to maintain soil moisture close to 80% of field capacity throughout the pot culture period. Two harvests were with consecutive resowing to maintain a constant number of plants. Ryegrass leaf harvests were performed 28 d after emergence, and the roots were cut to 0.5–1 cm segments and then returned to the soil prior to re-potting. A total of 12 crops of ryegrass were harvested with the treatments of biotite, illite and vermiculite; while 9 crops of ryegrass were harvested for the other three mineral treatments.

The DM yield of each crop of ryegrass was determined after the leaves were oven-dried to a constant weight. Ryegrass leaves were digested with H_2_SO_4_–H_2_O_2_ for K determination^42^. Plant K uptake was computed from K concentrations in the plant and DM yield^43^. Cumulative K uptake was the summation of plant uptake in each crop harvest.

### Statistical Analyses

All data are means of three or four values. Significant differences between treatments were tested through analysis of variance (*P* < 0.05). Treatments were compared with the control through the least significant difference test, using the SPSS 19.0. A simple linear correlation and a nonlinear regression between variables were calculated using Linear and Nonlinear Regression of Sigmaplot 12.0, respectively.

## Author Contributions

H.Y.W., T.L. and J.W. initiated and designed the research, J.W. and Z.J.Z. performed the experiments, T.L. and H.Y.W. analysed the data and wrote the paper, J.M.Z. and Z.J.Z. also revised and edited the manuscript.

## Figures and Tables

**Table 1:** Basic properties for the tested K-bearing phyllosilicate minerals

Mineral type	Chemical composition as analyzed by XRF (%, w/w)									Slow available K (mg kg^−1^)	CEC (cmol kg^−1^)
	SiO_2_	Al_2_O_3_	Fe_2_O_3_	TiO_2_	MgO	CaO	Na_2_O	K_2_O	P_2_O_5_		
Biotite	42.37	16.71	9.99	0.56	20.38	0.89	0.44	6.73	0.25	10399	7.25
Muscovite	53.09	28.40	5.87	0.74	0.97	0.04	0.49	9.18	0.07	345	2.30
Phlogopite	49.57	28.42	5.82	0.75	1.07	0.11	0.52	10.02	0.12	779	1.75
Illite	46.89	37.51	0.32	1.62	0.12	0.11	0.06	9.68	0.06	865	6.00
Vermiculite	68.94	13.15	2.25	0.18	2.10	1.62	1.13	2.26	0.14	521	47.05
Smectite	54.2	14.29	10.47	1.04	5.88	1.01	1.2	3.92	0.27	5237	14.25

**Table 2:** Rate constant of equations (g kg^−1^ h^−1^), coefficient of determination (R^2^) and standard error of the estimate (g kg^−1^) for K release from K-bearing phyllosilicate minerals by 0.2 M NaTPB

Time (h)	Mineral types	First-order equation			Parabolic diffusion equation			Zero-order equation			Elovich equation			Power function equation		
		Rate constant	R^2^	SE	Rate constant	R^2^	SE	Rate constant	R^2^	SE	Rate constant	R^2^	SE	Rate constant	R^2^	SE
0–2	Biotite	18.401	0.962	1.04	11.603	0.907	1.61	6.126	0.766	2.55	4.353	0.991	0.49	16.48	0.949	1.20
	Muscovite	0.622	0.372	0.07	0.211	0.995	0.01	0.121	0.979	0.01	0.073	0.912	0.03	0.638	0.944	0.02
	Phlogopite	0.473	0.827	0.05	0.287	0.985	0.02	0.158	0.906	0.04	0.102	0.973	0.02	0.422	0.994	0.01
	Illite	4.176	0.844	0.47	2.734	0.994	0.09	1.557	0.977	0.18	0.945	0.922	0.33	3.239	0.988	0.13
	Vermiculite	9.932	0.991	0.21	5.247	0.982	0.31	3.025	0.989	0.24	1.777	0.875	0.81	3.954	0.996	0.15
	Smectite	0.987	0.557	0.07	0.233	0.969	0.02	0.127	0.868	0.04	0.085	0.991	0.01	1.002	0.996	0.01
2–96	Biotite	47.715	0.959	3.87	3.405	0.896	4.39	0.267	0.784	6.31	8.83	0.964	2.59	17.569	0.941	3.31
	Muscovite	1.536	0.898	0.19	0.117	0.996	0.03	0.01	0.952	0.10	0.289	0.982	0.06	0.543	0.998	0.02
	Phlogopite	8.893	0.988	0.33	0.572	0.974	0.35	0.049	0.997	0.04	1.366	0.888	0.73	0.169	0.992	0.19
	Illite	19.086	0.934	2.40	1.765	0.997	0.39	0.146	0.967	1.21	4.363	0.974	1.08	4.529	0.997	0.40
	Vermiculite	21.566	0.967	1.91	1.971	0.981	1.03	0.16	0.917	2.16	4.943	0.987	0.84	5.07	0.992	0.69
	Smectite	1.271	0.856	0.05	0.021	0.767	0.04	0.002	0.643	0.05	0.056	0.896	0.03	1.083	0.886	0.03

**Table 3:** Correlation coefficients between K release characteristics and plant indices

Index	Cumulative DM yield	Cumulative K uptake	Average relative DM yield	Average relative K uptake
K release amount (0–2 h)	0.963**	0.947*	0.968**	0.930*
K release amount (2–96 h)	0.946*	0.925*	0.962**	0.885*
Rate constant of power function equation (0–2 h)	0.950*	0.909*	0.950**	0.865
Rate constant of power function equation (2–96 h)	0.929*	0.879*	0.927*	0.830
Slow available K	0.575	0.477	0.563	0.395
Total K	−0.726	−0.704	−0.677	−0.699

*, ** Significant at 0.05 and 0.01 levels, respectively.

**Table 4:** Kinetic equations used to describe K-release from phyllosilicate minerals by 0.2 M NaTPB

Kinetic equation	Expression form
First – order equation	ln(*C*_0_ − *C_t_*) = ln*C*_0_ − *K_d_t*
Parabolic diffusion equation	*C_t_*/*C*_0_ = *a* + *bt*^1/2^
Zero-order equation	*C_t_* = *a* + *b*ln*t*
Elovich equation	*C_t_* = *a* + *b*ln*t*
Power function equation	*Y* = *at^b^*

*C*_0_ is the maximum desorbable K (it was found from the equilibrium plots of K release versus time) in g kg^−1^; *C*_t_ is the K released after any time period; *K*_d_ is the apparent release-rate coefficient (h^−1^) and was calculated from the slope of each first-order plot; *t* is time (h); *a* and *b* are constants; *Y* is the quantity of K released at time *t*.
